# The effect of broccoli stem extract cream (*Brassica oleracea* L.) on macular scars post-acne

**DOI:** 10.3389/fmed.2025.1680933

**Published:** 2025-11-20

**Authors:** Fitriend Syahputri, Imam Budi Putra, Nelva Karmila Jusuf

**Affiliations:** 1 Faculty of Medicine, Dermatology, Venereology, and Aesthetic Study Program, Universitas Sumatera Utara, Medan, Indonesia; 2 Faculty of Medicine, Department of Dermatology and Venereology, Universitas Sumatera Utara, Medan, Indonesia; 3 Prof. Dr. Chairuddin Panusunan Lubis Universitas Sumatera Utara Hospital, Medan, Indonesia

**Keywords:** macular scar, acne vulgaris, broccoli stem extract, herbal treatment, *Brassica oleracea* L.

## Abstract

**Introduction:**

Post-acne macular scars often cause skin discoloration, affecting appearance and self-confidence. To address this issue, various topical therapies have been developed. Broccoli (*Brassica oleracea* L.) is widely cultivated in North Sumatra. Broccoli stem, typically discarded as a byproduct, represents a sustainable resource that can be repurposed for skincare applications. Broccoli stem extract contains bioactive constituents, including sulforaphane and flavonoids, which exhibit antioxidant and anti-inflammatory activities. These properties suggest its potential role in improving post-acne macular scars.

**Objectives:**

To analyse the effects of 6% broccoli stem extract cream (*Brassica oleracea* L.) on post-acne macular scars.

**Methods:**

This quasi-experimental pretest-posttest study included 30 male and female subjects aged ≥18 years with post-acne macular scars, selected according to inclusion and exclusion criteria. Baseline data, melanin index, and erythema index were assessed before and after applying 6% broccoli stem extract cream for 8 weeks. The Wilcoxon signed-rank test was used for data analysis, with statistical significance set at *p* < 0.05.

**Results:**

Most subjects were women in the late adolescent age group (18–25 years). Statistically significant reductions (*p* < 0.001) were observed in both the melanin index and erythema index after 8 weeks. Clinical improvement was evident, and no adverse effects were reported, highlighting the cream’s safety for sensitive post-inflammatory skin. All subjects (100%) reported satisfaction with the cream.

**Conclusion:**

The use of 6% broccoli stem extract cream effectively improved pigmentation and reduced melanin and erythema indices in post-acne macular scars. Its good tolerability and high patient satisfaction support its potential as a natural, safe alternative to conventional topical therapies.

## 1 Introduction

Acne scarring occurs as a consequence of aberrant wound healing, typically resulting from injury to the pilosebaceous unit and inflammation of the surrounding tissue during the acne resolution phase (1). Macular acne scars are flat erythematous, hyperpigmented, or hypopigmented lesions (2). Management for macular acne scars can be given topical therapy containing tretinoin, niacinamide and also herbal ingredients. Broccoli (*Brassica oleracea* L.) contains non-enzymatic bioactive substances, including antioxidants such as ascorbic acid (vitamin C), vitamin E, carotenoids, phenolic compounds, especially flavonoids and polyphenols which exhibit antioxidant, anti-inflammatory, anti-melanogenic, wound healing, and skin lightening effects (3–6). By utilizing broccoli stems, which are often discarded, this study explores a sustainable approach to acne scar treatment while leveraging the plant’s bioactive properties (Table 1).

**Table 1:** Distribution subject based on gender and age group.

Subject characteristics	Macular post acne scar (*n* = 30)	
	*n*	%
Gender		
Male	13	43.3
Female	17	56.7
Age group		
18–25 years	16	53.3
26–35 years	11	36.7
36–45 years	3	10

## 2 Methods

### 2.1 Patients and study design

This quasi experimental clinical study was conducted using a pretest-posttest design on 30 patients aged 18 years and older who had been diagnosed with macular scar post-acne, and provided written informed consent. Most participants had Fitzpatrick skin type III–IV, which is typical in this population. Patients who were undergoing treatment for macular scars post acne, consuming antioxidants, pregnant, or breastfeeding were excluded. Participants who missed cream application for three consecutive days or applied for <7 weeks were considered dropouts. Clinical assessments as well as measurements of melanin and erythema index using Mexameter® MX 18 device (C&K, Courage-Khazaka, Köln, Germany) were performed at baseline and at weeks 2, 4, 6, and 8 following the topical application of 6% broccoli stem extract cream. Any adverse effects observed during the study were recorded and evaluated at the conclusion of the treatment period. This pre-experimental clinical study was conducted using a pretest-posttest design on 30 patients aged 18 years and older with post-acne macular scars who provided written informed consent. Most participants had Fitzpatrick skin type III–IV, which is typical in this population. Ethical approval for this research was granted by the Health Research Ethics Committee of the Faculty of Medicine, Universitas Sumatera Utara (1,202/KEPK/USU/2024). Statistical analysis was performed using the Wilcoxon signed-rank test, with significance at *p* < 0.05. The cream formulation contained 6% broccoli stem extract as the active ingredient with a standard cream base (water, emulsifying agents, stabilizers).

## 3 Results

Thirty participants were enrolled. Most were female (*n* = 17; 56.7%) and aged 18–25 years (*n* = 16; 53.3%). The mean melanin index decreased from 184.28 ± 88.65 at baseline to a mean of 89 (range: 9–289.67) after 8 weeks (*p* < 0.001), as shown in Table 2. The baseline erythema index was 378.52 ± 75.29, decreasing to 242.28 ± 71.75 (*p* < 0.001) (Table 3). All patients improved in melanin and erythema indices, with individual variation in response time. No adverse reactions were reported, emphasizing the cream’s tolerability.

**Table 2:** Evaluation macular scar post acne based on melanin index.

Period	*n*	Melanin index (Mean ± SD)	*p*-value
Baseline	30	184.28 ± 88.65	<0.001*
2nd week	30	145.12 ± 92.45	
4th week	30	127.84 ± 85.37	
6th week	30	105.50 ± 80.12	
8th week	30	89.00 ± 72.45	

**Table 3:** Evaluation macular scar post acne based on erythema index.

Period	*n*	Erythema index (Mean ± SD)	*p*-value
Baseline	30	378.52 ± 75.29	<0.001*
2nd week	30	354.33 ± 73.28	
4th week	30	315.39 ± 74.15	
6th week	30	284.10 ± 74.55	
8th week	30	242.28 ± 71.75	

## 4 Discussion

This study shows that broccoli stem extract cream has a good effect in improving the appearance of subjects with macular scars post-acne. The broccoli stem extract cream significantly reduced the melanin and erythema index (*p* < 0.001). In addition, no side effects were observed in any of the subjects.

Macular scars are erythematous, hyperpigmented, or hypopigmented flat lesions that can last for months, impacting self-esteem and quality of life (2, 7–11). Their pathogenesis follows normal wound healing phases: haemostasis, inflammation, proliferation, and remodelling (12–14).

The pathogenesis of macular acne scars follows phases of normal wound healing, including haemostasis, inflammation, proliferation, and remodelling. The haemostasis phase occurs immediately after damage to the dermis and triggers the release of cytokines, neutrophils, and macrophages as inflammation begins (12, 13). During the inflammatory phase, vasodilation and increased vascular permeability occur as a result of mediators such kinins, histamine, prostaglandins, and leukotrienes. These changes lead to the appearance of erythema and edema in affected skin. Vasodilation and erythema replace the initial vasoconstriction, and there is an increase in melanogenesis, which contributes to post-inflammatory erythema and hyperpigmentation following acne-induced skin injury (12, 14).

The proliferative phase involves an increase in fibroblast activity, granulation tissue formation, and collagen production. During the remodelling phase, granulation tissue is gradually substituted with fibrous connective tissue. Myofibroblasts within the wound demonstrate contractile abilities facilitated by actin filaments and establish gap junctions with fibronectin and collagen. Their contraction draws the wound margins closer, resulting in wound contraction and a decrease in scar size (13, 14).

The effective treatment of macular scars remains a clinical challenge. Current therapeutic options include topical agents such as tretinoin and adapalene, as well as chemical peels and laser therapies that target vascular and pigmentary components (15, 16). Recent interest has also emerged in botanical or plant-based therapies for dermocosmetics, including antioxidants and natural anti-inflammatory compounds with fewer side effects (3, 17).

The stem of broccoli contains dietary fiber, vitamins A and C, glucosinolates, sulforaphane, selenium, and flavonoids. Pectin, vitamin C, flavonoids, and sulforaphane contribute synergistically to antioxidant, anti-inflammatory, and anti-melanogenic effects, supporting skin repair and pigmentation improvement (17–20).

One of the key flavonoids, quercetin, offers potent antioxidant and anti-inflammatory effects, helping accelerate wound healing by reducing oxidative stress and limiting both acute and chronic inflammation (21). Pectin, a polysaccharide in broccoli cell walls containing galacturonic acid, may inhibit melanin production and acts as a skin-brightening agent. During acne wound healing, inflammation can trigger uneven melanin production, leading to hyperpigmentation (22, 23). Vitamin C in broccoli supports collagen synthesis as a cofactor for hydroxylase enzymes, crucial for forming stable collagen structures that maintain tissue strength and repair during wound healing (17). Collectively, quercetin, pectin, and vitamin C act synergistically with other bioactive compounds such as sulforaphane to reduce oxidative stress, limit inflammation, suppress melanogenesis, and support collagen-mediated skin repair, which likely underlies the clinical improvements and high patient satisfaction observed in this study.

In this study, patients treated with 6% broccoli stem extract cream showed visible improvements in skin tone uniformity and reduction in pigmentation intensity. This effect is attributed to the multiple bioactive compounds in broccoli stem extract. Inflammation caused by acne increases oxidative stress, stimulating melanocyte activity and melanin production (24). Flavonoids and polyphenols act as potent antioxidants, neutralizing free radicals, reducing inflammation, and preventing further hyperpigmentation. Sulforaphane, a key bioactive compound, inhibits tyrosinase activity, a critical enzyme in melanin synthesis (25). Previous studies support these mechanisms: Jusuf et al. reported that antioxidants from broccoli flower extract inhibited the MAPK pathway, reduced MMP-1 expression, and increased type I procollagen, suggesting anti-photoaging potential (26, 27). Jusuf and Putra demonstrated that a 3% broccoli flower extract cream was safe and effective for melasma (6). Marlina, Pangkahila, and Wiraguna showed that topical broccoli extract in UV-B–exposed guinea pigs significantly lowered melanin levels (28).

Polyphenolic compounds, including flavonoids, exert anti-inflammatory effects by modulating gene expression and inhibiting skin-degrading enzymes such as hyaluronidase, collagenase, and elastase, supporting skin repair. Sulforaphane enhances this by reducing oxidative stress and regulating inflammation (29). Collectively, these compounds act synergistically—antioxidant, anti-inflammatory, and anti-melanogenic effects converge to reduce erythema and pigmentation while promoting collagen-mediated skin repair. This multi-targeted mechanism likely underlies the observed clinical improvements and high patient satisfaction in this study.

Moreover, the anti-inflammatory effects of broccoli-derived phytochemicals may help reduce residual inflammation that sustains macular scars post-acne. The presence of vitamin C in the extract may also contribute to inhibiting melanin synthesis and promoting collagen production, which supports skin repair (3, 6).

The beneficial effects of 6% broccoli stem extract cream on post-acne macular scars can be attributed to multiple bioactive compounds with complementary and synergistic mechanisms: flavonoids and polyphenols act as potent antioxidants that neutralize reactive oxygen species (ROS), reducing oxidative stress-induced melanogenesis and inflammation (20); sulforaphane inhibits tyrosinase activity and downregulates its gene expression to decrease melanin synthesis (25); vitamin C and selenium serve as essential cofactors for collagen synthesis, enhancing structural integrity and tissue healing (17); and pectin reduces melanin production via *α*-MSH/TRY modulation (23).

Collectively, these antioxidant, anti-inflammatory, and anti-melanogenic activities likely underlie the observed clinical improvements and high patient satisfaction. This multi-targeted action highlights the potential of broccoli stem extract as a safe and effective alternative for managing post-acne macular scars.

Limitations of this study include a small sample size, short treatment duration, and the absence of comparative trials with standard agents such as niacinamide or retinoids. Future studies should involve larger populations, extended follow-up periods, and head-to-head comparisons with established therapies to confirm efficacy and position broccoli stem extract within the therapeutic hierarchy.

## 5 Conclusion

The 6% broccoli stem extract cream significantly improved post-acne macular scars by reducing melanin and erythema indices. Without causing adverse effects and with high patient satisfaction. These findings highlight its potential as a safe, natural alternative for acne-related hyperpigmentation. Future studies should compare it with standard therapies and evaluate long-term outcomes.

## Data Availability

The original contributions presented in the study are included in the article/supplementary material. Further inquiries can be directed to the corresponding author.
